# Carpal Tunnel Syndrome and Ulnar Nerve Entrapment at the Elbow Are Not Associated With Plasma Levels of Caspase-3, Caspase-8 or HSP27

**DOI:** 10.3389/fnins.2022.809537

**Published:** 2022-03-04

**Authors:** Elin Bergsten, Mattias Rydberg, Lars B. Dahlin, Malin Zimmerman

**Affiliations:** ^1^Department of Orthopedics, Helsingborg Hospital, Helsingborg, Sweden; ^2^Department of Translational Medicine—Hand Surgery, Lund University, Lund, Sweden; ^3^Department of Hand Surgery, Skåne University Hospital, Malmö, Sweden; ^4^Department of Biomedical and Clinical Sciences, Linköping University, Linköping, Sweden

**Keywords:** carpal tunnel syndrome, ulnar nerve compression syndromes, caspase-3, caspase-8, HSP27, heat-shock proteins, apoptosis

## Abstract

**Background:**

Nerve compression disorders, such as carpal tunnel syndrome (CTS) and ulnar entrapment at the elbow (UNE), may be associated with apoptosis and neuroprotective mechanisms in the peripheral nerve that may be detected by biomarkers in the blood. The relationships between CTS and UNE and two biomarkers of apoptosis, i.e., caspase-3 and caspase-8, and the neuroprotective factor Heat Shock Protein 27 (HSP27) in plasma were examined in a population-based cohort.

**Method:**

The biomarkers caspase-3, caspase-8 and HSP27 were measured in plasma at inclusion of 4,284 study participants aged 46–68 years in the population-based Malmö Diet and Cancer study (MDCS). End-point retrieval was made from national registers concerning CTS and UNE. Independent *t*-test was used to examine the association between caspase-3, caspase-8 and HSP27 plasma levels and incidence of CTS and UNE. Cox proportional hazards regression was used to investigate if plasma levels of caspase-3, caspase-8 and HSP27 affected time to diagnosis of CTS or UNE.

**Results:**

During the mean follow-up time of 22 years, 189/4,284 (4%) participants were diagnosed with CTS and 42/4,284 (1%) were diagnosed with UNE. No associations were found between incident CTS or UNE and the biomarkers caspase-3, caspase-8 and HSP27 in plasma.

**Conclusion:**

The apoptotic biomarkers caspase-3 and caspase-8 and the neuroprotective factor HSP27 in plasma, factors conceivably related to a nerve injury, are not associated with the nerve compression disorders CTS and UNE in a general population.

## Introduction

Nerve compression disorders in the upper extremity, such as carpal tunnel syndrome (CTS) and ulnar nerve entrapment at the elbow (UNE), are common conditions. Symptoms of intermittent or permanent numbness, tingling, pain, and weakness, with or without muscle atrophy, in the affected hand have a negative impact on quality of life (Dawson, 1993; Atroshi, 2011). The pathophysiology is still not completely clarified (Rempel et al., 1999) and the use of biomarkers for support in diagnostic and severity evaluations may be an additional step toward better treatment.

Programmed cell death, apoptosis, of a variety of cell types in a peripheral nerve has been implicated in the pathophysiology of CTS and UNE. Chronic compression of a peripheral nerve in animal models cause activation, proliferation and apoptosis of Schwann cells (Gupta and Steward, 2003) with loss of myelin and subsequent remyelination; however, the new myelin is markedly thinner and has shorter internodal distances (Ochoa et al., 1972; Ludwin and Maitland, 1984). Compressed nerves in diabetic rats show an increased activation of Schwann cells in the nerve as well as of sensory neurons in dorsal root ganglia (DRG), indicating an increased susceptibility to compression among diabetic subjects (Dahlin et al., 2008). A reduction in the number of myelinated nerve fiber in an uncompressed nerve at the same level as the carpal tunnel is seen in subjects with CTS compared to control subjects, and is even more pronounced if the subject also has diabetes (Thomsen et al., 2009). These studies indicate that some subjects are more vulnerable to nerve compression and that mechanism may be even more pronounced in subject with diabetes. A more severe nerve injury, such as nerve transection and repair, also induces activation and apoptosis of Schwann cells; the latter being more pronounced in diabetic rats (Stenberg and Dahlin, 2014). Thus, there are indications that a nerve injury, and particularly nerve compression, may induce activation and apoptosis in the nerve trunk.

Apoptosis can follow different patterns of coordinated activation of caspases in a cascade of events. In the extrinsic pathway, caspase-8 is activated. Both the extrinsic and the intrinsic pathways activate the executioner caspase-3, which cleaves key structural proteins causing cell death (Slee et al., 2001; Elmore, 2007; Ponder and Boise, 2019). A nerve injury in an animal model leads to an increased intracellular expression of caspase-3 and caspase-8 in sensory neurons in DRG (Wiberg et al., 2018) as well as of caspase-3 in the injured nerve (Stenberg et al., 2012). The intracellular caspases are released into the extracellular matrix after the cell has died and can be measured in plasma (Xue et al., 2017). An association between plasma levels of caspase-8 and incidence of ischemic stroke (Muhammad et al., 2018) as well as coronary events (Xue et al., 2017) has been found, indicating that such proteins can be used as biomarkers for injury.

Small heat shock proteins (sHSP) protect cells from outer stress, such as toxic chemicals, oxidative stress and heat shock (Acunzo et al., 2012), and are detected in human nerve biopsies both from healthy and diabetic subjects (Ising et al., 2021). In particular, HSP27 plays a major role in inhibiting apoptosis (Hunt et al., 2012) and reduces ischemic damage following a stroke (Sharp et al., 2013; Teramoto et al., 2013). In subjects with diabetes, HSP27 protects sensory neurons from damage (Korngut et al., 2012), and higher plasma levels of HSP27 are associated with fewer signs of peripheral neuropathy, as well as with better nerve function (Pourhamidi et al., 2011). In accordance, it is possible that an increased expression of HSP27 may be present in plasma of subjects with a nerve compression disorder, such as CTS and UNE.

The present study aimed to investigate potential associations between plasma levels of caspase-3, caspase-8 as well as HSP27 and incidence of CTS and UNE.

## Subjects and Methods

### Study Population

The study participants are part of the cardiovascular cohort of the Malmö Diet and Cancer Study (MDCS-CC) (Berglund et al., 1993b). The Malmö Diet and Cancer Study (MDCS) cohort is a population-based cohort in Malmö that started in 1991. The city of Malmö at that time had 230,000 inhabitants and is located in the southeastern part of the southern region of Scania in Sweden. All individuals born between 1923-1945 (men) and 1923–1950 (women) and living in Malmö were sent letters of invitation or were included by spontaneously contacting the screening center. Participation rate was approximately 41% of the population of Malmö in the included age span. Cohort characteristics, methods and inclusion criteria for MDCS have been previously described (Berglund et al., 1993a; Hedblad et al., 2000; Manjer et al., 2001; Xue et al., 2017). Between March 1991 and September 1996, a total of 28,449 individuals (11,246 men and 17,203 women) underwent a baseline examination.

Between October 1991 and February 1994, 6,103 individuals, i.e., a random 50% of the MDCS participants at the time, were invited to the MDCS cardiovascular cohort (MDCS-CC). MDCS-CC was originally designed to study carotid artery disease and insulin resistance. The 5,540 subjects who accepted inclusion participated in a second visit for collection of fasting blood samples. All participants had their height and weight measured at study start. Body mass index (BMI) was calculated as [weight (kg)]/[height × height (m)]. Smoking, alcohol consumption and use of antihypertensive treatment was self-reported in a questionnaire. Alcohol consumption was then converted into grams of alcohol consumed per day (g/day). Blood pressure was measured at study start and hypertension was defined as having a systolic blood pressure (BP) ≥ 140 mm Hg or diastolic BP ≥ 90 mm Hg, measured in a supine position using a mercury column sphygmomanometer. Prevalent DM vas defined as either a self-reported physician’s diagnosis of DM, the use of anti-diabetic medication, or a fasting whole blood sugar > 6.0 mmol/L at baseline (Berglund et al., 1993b).

### Laboratory Analyses

The biomarkers were analyzed in fasting EDTA-plasma samples that had been stored in −80°C until analysis in 2015, as has been previously reported (Lind et al., 2021). After excluding 368 participants due to incomplete clinical data and another 307 participants with missing blood samples, the remaining 4,865 samples were sent for analysis; however, 52 samples did not pass the internal quality control for the biomarker analysis (Lind et al., 2021). Caspase-3 was measured using OLINK Proseek ^®^ Multiplex Oncology I96 × 96 Panel. Caspase-8 and HSP27 were measured in the OLINK Proseek Multiplex CVD I 96 × 96 Panel. The values are expressed as arbitrary units (AU) on a 2log scale. The details of the analysis procedure have been mentioned in detail elsewhere (Xue et al., 2017; Muhammad et al., 2018; Lind et al., 2021). The measuring range of caspase-3 (i.e., lower and upper limits of quantification) was 1.9--31,250 pg/ml. The coefficient of variation was 16% based on values over the limit of detection (LOD: 1.91 pg/ml). The coefficient of variation for caspase-8 was 22% based on linearized values over the limit of detection (LOD: 0.48 pg/ml)^1^.

### Included Participants

**FIGURE 1:**
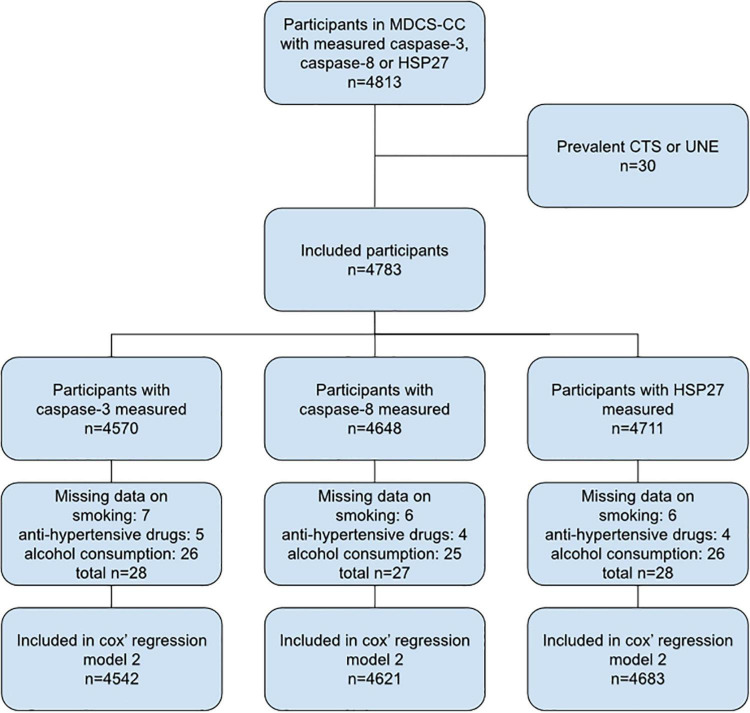
Flow chart of included study participants. MDCS-CC, The Malmö Diet and Cancer Study—Cardiovascular cohort; CTS, carpal tunnel syndrome; UNE, ulnar nerve entrapment at the elbow.

There were 4,813 participants in the MDCS-CC database with caspase-3, caspase-8 or HSP27 measured. At study start, 30 participants had previously been diagnosed with CTS and/or UNE and were therefore excluded. Finally, 4,783 participants were included in the present study (Figure 1).

### Data Collection

End point retrieval was performed by linking each study participant’s unique personal identification number to several Swedish national registers administered by the Swedish National Board of Health and Welfare, including the Inpatient register, from study start, the Day Surgery Register, from 1997, and the Specialized Outpatient Register, from 2000.

The CTS and UNE diagnoses were made by physicians in specialist care and were based on clinical findings alone or with the support of electroneurography (details about the basis for diagnosis are not available). Codes 352.02, 357.01, 354A and G56.0 from the International Classification of Disease (ICD) version 8, 9 and 10 were used to identify participants with CTS. Codes 352.01, 357.0, 354C and G56.2 from ICD 8, 9 and 10 were used to identify participants with UNE.

Study participants were followed from the baseline examination in 1992–1994 until first CTS or UNE diagnosis, death, emigration from Sweden, or until end of follow-up December 31st, 2018.

### Statistical Analysis

Descriptive statistics were used for clinical and demographic characteristics of the participants. Independent sample *t*-test was used to compare caspase-3, caspase-8 and HSP27 levels, between CTS and UNE groups and the groups without CTS or UNE. Cox proportional hazards regression models were used to examine associations between caspase-3, caspase-8 and HSP27 levels and incident CTS or UNE. Caspase-3, caspase-8 and HSP27 levels were divided into four quartiles and the lowest quartile was used as the reference category. Hazard ratios (HR) with 95% confidence intervals were calculated. Possible effect modification of age, sex, BMI, prevalent diabetes (DM), smoking, hypertension, use of hypertensive treatment and alcohol consumption (all at study start), with respect to the association between caspase-3, caspase-8 and HSP27 levels and incident CTS or UNE, were adjusted for in the multivariate model. Kaplan–Meier plots were used to illustrate the quartiles of caspase-3, caspase-8 and HSP27 in relation to incidence of CTS and UNE.

In all calculations, a *p*-value < 0.01 was considered significant. IBM SPSS Statistics 25 (Armonk, NY, United States) was used for the statistical analysis.

## Results

In total, 2,874/4,783 (60%) women and 1,909/4,783 (40%) men were included in the study. At baseline, participants were between 46 and 68 years of age, and mean age was 57 (SD ± 5.9) years. BMI at study start varied between 15.2 and 50.7, with a mean of 25.6 (SD ± 3.9), and 197/4,783 (4%) participants had prevalent DM.

**FIGURE 2:**
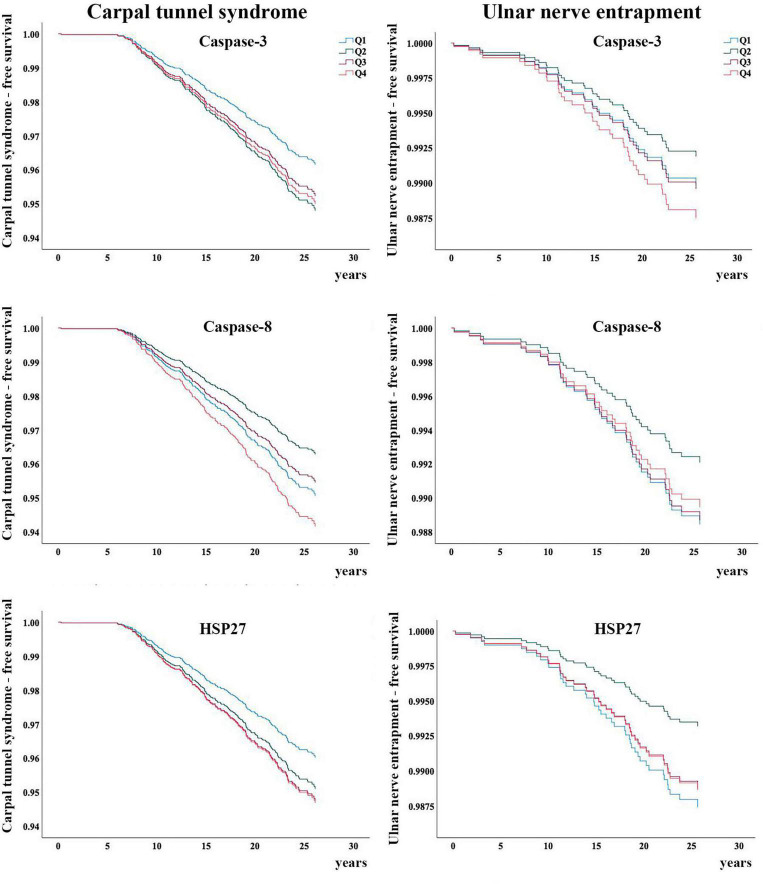
Carpal tunnel syndrome-free and ulnar nerve entrapment-free survival in relation to the quartiles of caspase-3, caspase-8 and HSP27, respectively. Q, quartiles.

During the follow-up period (1991–1996 to 2018; mean 22 years, SD ± 6), 189/4,783 (4%) participants were diagnosed with CTS and 42/4,783 (1%) participants were diagnosed with UNE. Of these, 16 participants were diagnosed with both CTS and UNE during the study period. Baseline characteristics are presented in Table 1. Participants who were diagnosed with either CTS or UNE did not have higher levels of caspase-3, caspase-8 or HSP27 at inclusion compared to participants who were not diagnosed with CTS or UNE (Table 2). No associations between levels of caspase-3, caspase-8 or HSP27 with either CTS nor UNE were found, this was unaffected by adjustment for risk factors (Tables 3, 4). BMI, age, smoking, hypertension, use of hypertensive drugs, alcohol consumption and prevalent DM did not differ across quartiles of caspase-3, caspase-8 and HSP27 (data not shown). Kaplan–Meier plots of CTS-free and UNE-free survival in relation to quartiles of caspase-3, caspase-8 and HSP27 are shown in Figure 2.

**TABLE 1:** Baseline characteristics of study participants.

	No CTS or UNE (*n* = 4,568)	Incident CTS (n = 189)	Incident UNE (n = 42)
Women n (%)	2,731 (60)	128 (68)	24 (57)
BMI (mean ± SD)	25.6 ± 3.9	26.6 ± 4.0	25.3 ± 3.4
Smoking habits n (%)	1,119 (26)	42 (22)	11 (26)
Hypertension n (%)	2,110 (46)	78 (41)	16 (38)
Antihypertensive medication n (%)	763 (17)	27 (14)	5 (12)
Age (years; mean ± SD)	57.5 ± 6.0	57 ± 5.7	56.1 ± 5.3
Alcohol g/day (mean ± SD)	10.1 ± 12.0	9.5 ± 10.9	8.9 ± 8.7
Prevalent DM n (%)	178 (4)	18 (10)	2 (5)

*BMI, body mass index (body mass in kilograms divided by square of body height in meters); CTS, carpal tunnel syndrome; DM, diabetes mellitus; SD, standard deviation; UNE, ulnar nerve entrapment at the elbow; NB, 16 participants were diagnosed with both CTS and UNE.*

**TABLE 2:** Levels of caspase-3, caspase-8 or HSP27 and incidence of CTS and UNE.

	Incident CTS or UNE	No CTS or UNE	*P*-value
**CTS**			
Caspase-3	10.7 SD ± 1.0 (*n* = 176)	10.7 SD ± 1.0 (*n* = 4,570)	0.74
Caspase-8	1.6 SD ± 0.7 (*n* = 181)	1.5 SD ± 0.7 (*n* = 4,648)	0.41
HSP27	4.8 SD ± 0.8 (*n* = 187)	4.7 SD ± 0.9 (*n* = 4,711)	0.32
**UNE**			
Caspase-3	10.7 SD ± 1.0 (*n* = 40)	10.7 SD ± 1.0 (*n* = 4,570)	0.98
Caspase-8	1.5 SD ± 0.8 (*n* = 41)	1.5 SD ± 0.7 (*n* = 4,648)	0.99
HSP27	4.7 SD ± 0.9 (*n* = 42)	4.7 SD ± 0.9 (*n* = 4,711)	0.97

*All values presented as mean level of AU: arbitrary units. CTS, carpal tunnel syndrome; SD, standard deviation; UNE, ulnar nerve entrapment at the elbow. Statistical analysis 2-tailed t-test.*

**TABLE 3:** Incidence of carpal tunnel syndrome in relation to quartiles of caspase-3, caspase-8 and HSP27.

CTS	Q1 (reference)	Q2	Q3	Q4	HR per SD
**Caspase-3**					
No of participants	1,142	1,137	1,146	1,145	
Incidence of CTS	37	50	44	45	
Model 1	1	0.77 (0.50–1.19) *p* = 0.23	1.04 (0.69–1.56) *p* = 0.84	0.95 (0.63–1.45) *p* = 0.82	1.06 (0.91–1.23) *p* = 0.47
Model 2	1	0.77 (0.50–1.19) *p* = 0.24	1.06 (0.71–1.59) *p* = 0.79	0.95 (0.63–1.45) *p* = 0.82	1.05 (0.90–1.22) *p* = 0.54
**Caspase-8**					
No of participants	1,158	1,164	1,163	1,163	
Incidence of CTS	50	36	43	52	
Model 1	1	0.84 (0.57–1.25) *p* = 0.40	0.63 (0.41–0.97) *p* = 0.03	0.77 (0.52–1.16) *p* = 0.22	1.12 (0.97–1.30) *p* = 0.12
Model 2	1	0.90 (0.60–1.33) *p* = 0.59	0.66 (0.43–1.01) *p* = 0.06	0.79 (0.53–1.19) *p* = 0.27	1.09 (0.94–1.26) *p* = 0.26
**HSP27**					
No of participants	1,178	1,178	1,175	1,180	
Incidence of CTS	39	48	50	50	
Model 1	1	0.75 (0.49–1.13) *p* = 0.17	0.92 (0.62–1.37) *p* = 0.69	0.99 (0.67–1.47) *p* = 0.96	1.10 (0.94–1.27) *p* = 0.23
Model 2	1	0.77 (0.51–1.18) *p* = 0.24	0.94 (0.63–1.40) *p* = 0.76	1.02 (0.69–1.51) *p* = 0.92	1.08 (0.93–1.27) *p* = 0.29

*Model 1: HRs with 95% CIs, adjusted for age and sex.*

*Model 2: HRs (95% CI) adjusted for age, sex, smoking habits, use of alcohol, body mass index, prevalent diabetes mellitus, hypertension, and blood pressure-lowering medication.*

*HR, hazard ratio; CTS, carpal tunnel syndrome; SD, standard deviation.*

**TABLE 4:** Incidence of ulnar nerve entrapment at the elbow (UNE) in relation to quartiles of caspase-3, caspase-8 and HSP27.

UNE	Q1 (reference)	Q2	Q3	Q4	HR per SD
**Caspase-3**					
No of participants	1,142	1,137	1,146	1,145	
Incidence of UNE	10	8	10	12	
Model 1	1	0.81 (0.35–1.88) *p* = 0.62	0.65 (0.26–1.59) *p* = 0.34	0.83 (0.36–1.93) *p* = 0.67	1.03 (0.75–1.40) *p* = 0.90
Model 2	1	0.78 (0.33–1.81) *p* = 0.56	0.63 (0.26–1.55) *p* = 0.31	0.82 (0.35–1.89) *p* = 0.64	1.03 (0.75–1.42) *p* = 0.85
**Caspase-8**					
No of participants	1,158	1,164	1,163	1,163	
Incidence of UNE	12	8	11	10	
Model 1	1	1.10 (0.47–2.58) *p* = 0.83	0.75 (0.29–1.91) *p* = 0.55	1.07 (0.45–2.54) *p* = 0.87	1.04 (0.76–1.42) *p* = 0.81
Model 2	1	1.06 (0.50–2.51) *p* = 0.89	0.72 (0.28–1.84) *p* = 0.49	1.06 (0.45–2.51) *p* = 0.89	1.06 (0.77–1.45) *p* = 0.74
**HSP27**					
No of participants	1,178	1,178	1,175	1,180	
Incidence of UNE	13	7	11	11	
Model 1	1	1.11 (0.50–2.48) *p* = 0.80	0.60 (0.23–1.55) *p* = 0.29	0.99 (0.43–2.28) *p* = 0.98	1.03 (0.76–1.40) *p* = 0.84
Model 2	1	1.08 (0.48–2.42) *p* = 0.86	0.58 (0.22–1.50) *p* = 0.26	0.97 (0.42–2.24) *p* = 0.94	1.04 (0.77–1.42) *p* = 0.78

*Model 1: HRs with 95% CIs, adjusted for age and sex.*

*Model 2: HRs (95% CI) adjusted for age, sex, smoking habits, use of alcohol, body mass index, prevalent diabetes mellitus, hypertension, and blood pressure-lowering medication.*

*HR, hazard ratio; SD, standard deviation; UNE, ulnar nerve entrapment at the elbow.*

## Discussion

The present study indicates that there are no associations between diagnosis of CTS or UNE and plasma levels of caspase-3, caspase-8 or HSP27 during a long-time follow-up. Nerve compression disorders, such as CTS and UNE, may induce functional and structural alterations in the peripheral nerve as well as in the neurons in DRG and spinal cord depending on the severity and duration of the compression trauma. Initially, disturbances in the intraneural microcirculation, with subsequent edema formation, are induced. While the symptoms may not be constant, they may prompt patients to seek health care and get diagnosed, and may require decompression surgery. At the early stage, there are probably no structural changes, such as demyelination related to Schwann cells, degeneration of axons or pronounced changes in the nerve cell bodies; the latter with the purpose of transforming the function of the neuron from transferring impulses to structural repair and regeneration (Rempel et al., 1999). However, at later stages, with more severe compression trauma, intracellular alterations are induced in the Schwann cells and axonal transport in the neurons is inhibited with subsequent repair processes in the affected neurons. Activation of Schwann cells and neurons can be detected by expression of activating transcription factor 3 (ATF-3) (Pettersson et al., 2004; Isacsson et al., 2005; Dahlin et al., 2008), which is connected to neuroprotective mechanisms, such as HSP27. The amount of ATF-3 is also related to, and counterbalanced by, expression of apoptotic markers, such as caspase-3 (Gupta and Steward, 2003; Dahlin et al., 2008). The present data indicate that CTS and UNE are not associated with higher plasma levels of caspase-3 or caspase-8, which is probably explained by an insufficient compression leading to no, or a too low, induction of activation and apoptosis in the Schwann cells and in the neurons, where also the timing of evaluation in relation to the nerve compression is crucial. In rat models, a slight chronic compression induces a limited amount of caspase-3 presented in the nerve (Stenberg and Dahlin, 2014; Meyer et al., 2016), which is in accordance with the present findings of no increased plasma levels of caspase-3 and caspase-8. Thus, even though plasma levels of caspase-3 and caspase-8 indeed reflect apoptosis (Xue et al., 2017), a nerve injury caused by compression in CTS and UNE, is most likely not sufficient to be reflected in increased plasma levels of the evaluated biomarkers. In this study, the first incident of CTS and UNE was more than 5 years after the plasma sample and study start, and most cases were diagnosed after 10–15 years. The time lapse from possible apoptotic events in CTS or in UNE and onset of symptoms and the following time lapse from onset of symptoms to diagnosis is unknown. Thus, we do not know when in the CTS or UNE events the measurement of biomarkers was made. Most probably, measurements of caspase-3, caspase-8 and HSP27 made in connection with a more severe nerve injury, such as a nerve transection, or in a timelier manner to the compression trauma, will reveal increased levels, as shown in experimental studies (Stenberg and Dahlin, 2014; Meyer et al., 2016; Stenberg et al., 2016, 2017; Hazer Rosberg et al., 2021).

Plasma levels of HSP27, a protein associated to neuroprotection (Stenberg et al., 2017), have previously been studied in association with carotid artery atherosclerosis and atherosclerotic outcomes (Lind et al., 2021), as well as in patients with diabetes (Pourhamidi et al., 2011, 2014; Zimmerman et al., 2017) and related to diabetic neuropathy, which is another type of prolonged and general affection on the human body and particularly a peripheral nerve. Diabetic neuropathy, in which the subjects may also be affected with an additional nerve compression disorder, is probably a continuous ongoing event, particularly in type 1 diabetes, which leads to ongoing and permanent apoptotic and neuroprotective mechanisms with leakage of the biomarkers caspase-3, caspase-8 and HSP27 into plasma. Other events, such as ischemic stroke (Muhammad et al., 2018) and coronary events (Xue et al., 2017), are also known to be associated with higher levels of caspase-8. To conclude, the present limited injury to the peripheral nervous system, i.e., CTS or UNE, is not enough to significantly affect levels of apoptotic biomarkers in plasma compared to larger, general and prolonged events, such a diabetic neuropathy (Pourhamidi et al., 2011, 2014).

The strength of this study is its large, prospective, and population-based study design with a long follow-up period. The CTS and UNE cases were obtained through linkage with national registers. However, CTS and UNE diagnosed only in a primary care setting were not identified and thus not included. Such cases are most likely at earlier stages, not requiring surgery, and hence the peripheral nerve trauma should be minor. Such cases would most certainly not show any increased plasma levels of the presently used biomarkers. A limitation of the study is the lack of serial measurements to assess changes in biomarkers and risk factors over time, and that the time lapse from biomarker measurement to diagnosis is long. The population of the cohort is elderly (the youngest participant was 46 years old at study start), and nerve compression disorders, such as CTS and UNE, usually occur at 45–65 years of age (Atroshi, 2011; Anker et al., 2018; Giöstad and Nyman, 2019). The peak incidence of CTS in a general population in Sweden is in individuals aged 45–54 years (Atroshi et al., 1999; Atroshi, 2011), and the corresponding incidence of UNE in an American National Database is in individuals aged 61–65 years (Osei et al., 2017). This indicates that the population is appropriate to investigate. The study includes the important risk factors for CTS and UNE, such as age, female gender, hypertension, diabetes and obesity. However, data on other risk factors, like distal radius fractures, hypothyroidism, rheumatoid arthritis, exposure to vibrating hand held tools or repetitive wrist/elbow movements, were not available for analysis in the cohort.

In conclusion, the present study did not demonstrate any association between the nerve compression disorders CTS and UNE and plasma levels of peripheral nervous tissue biomarkers, i.e., caspase-3, caspase-8 and HSP27, in a general population followed for a mean of 22 years. Further studies are needed to increase knowledge of the role and timing of neuroprotection and apoptosis in CTS and UNE and any potential role of biomarkers in diagnosis and prognosis after surgical treatment.

## Data Availability Statement

The datasets presented in this article are not readily available because public access to data is restricted to Swedish Authorities (Public Access to Information and Secrecy Act), but data can be available for researchers after a special review that includes approval of the research project by both an Ethics Committee and the Authorities’ Data Safety Committees.

## Ethics Statement

The studies involving human participants were reviewed and approved by Regional Ethical Review Board, Lund, Sweden (LU51/90). The patients/participants provided their written informed consent to participate in this study.

## Author Contributions

LD designed the study. MR, LD, and MZ contributed to acquisition and interpretation of the data from the MDCS-CC Database and the Inpatient Register, the Day Surgery Register, and the Specialized Outpatient Register. EB did the calculations and wrote the draft of the manuscript. All authors contributed to data interpretation, article draft, revision of the manuscript and approved the final version of the manuscript.

## Conflict of Interest

The authors declare that the research was conducted in the absence of any commercial or financial relationships that could be construed as a potential conflict of interest.

## Publisher’s Note

All claims expressed in this article are solely those of the authors and do not necessarily represent those of their affiliated organizations, or those of the publisher, the editors and the reviewers. Any product that may be evaluated in this article, or claim that may be made by its manufacturer, is not guaranteed or endorsed by the publisher.

## Footnotes

1
www.olink.se
